# Evaluation of Lipid Metabolism and Nutritional Status in Male Goalball Players

**DOI:** 10.1515/hukin-2015-0100

**Published:** 2015-01-12

**Authors:** Krystyna Gawlik, Anna Zwierzchowska, Barbara Rosołek

**Affiliations:** 1 Posture Correction Department, The Jerzy Kukuczka Academy of Physical Education in Katowice; 2 Special Physical Education Department, The Jerzy Kukuczka Academy of Physical Education in Katowice

**Keywords:** lipid metabolism, nutrition, goalball

## Abstract

Lipid disorders, obesity and overweight are considered one of the most important modifiable cardiovascular risk factors. Population surveys carried out in Poland have demonstrated a tendency for lipid disorders to occur in 70% and overweight and obesity in more than half of Poles. No such studies have been conducted in groups of people with vision impairment so far. Yet, regular involvement of visually impaired people in sports is likely to reduce cardiovascular risk. Therefore, the authors attempted to evaluate the lipid profile and nutritional status of male goalball players. Thirty two blind or visually impaired male goalball players aged 20 to 45 years participated in the study during which somatic variables (BH, BM, WC, VFR, BMI) and the lipid profile (TC, LDL, HDL, TG) were evaluated. Overweight was found in 40.6% of athletes, with obesity being at the level of 9.3%. A high correlation was found between visceral fat and the BMI (r=0.7; p<0.001), as well as between visceral fat and WC (r=0.8; p<0.001). Abnormal total cholesterol levels were recorded for LDL (22% of study participants), HDL (17%) and triglycerides (13%). Lower levels of individual components of lipid profiles (and higher levels for HDL) were found in athletes with a normal BMI. A correlation was found between the BMI and TG (r=0.4, p<0.01), WC and TG (r=0.4, p<0.01), VFR and LDL ( r=0.4, p<0.05) and TG (r=0.5, p<0.001). The percentage of overweight and obese subjects with vision impairment was lower compared to the general population of men in Poland, with a more beneficial lipid profile. Regular physical activity of the study participants is likely to have a positive effect on their health.

## Introduction

Cardiovascular diseases are the leading cause of premature deaths or reduced quality of life in both Poland and worldwide (Alwan, 2011). Three groups of risk factors for cardiovascular diseases have been identified, including modifiable and non-modifiable risk factors, as well as so-called new cardiovascular risk markers and factors (Backer et al., 2003; Podolec et al., 2007; Undas et al., 2007). Among the modifiable risk factors, the most serious are lipid disorders, obesity and overweight (Mendis, 2011). An excess of body mass, waist circumference, and visceral fat rating have been found to be almost linearly correlated with prevalence of myocardial infarction and cerebrovascular accident (Grundy, 2002; McGill et al., 2002; Bhan et al., 2010). It has also been demonstrated that lower lipid levels reduce cardiovascular risk more than any other intervention (Heart Protection Study Collaborative Group, 2002). This process is attributable to proper diets and regular physical activity, which also play a key role in combating obesity and overweight. Population surveys by NATPOL PLUS and WOBASZ carried out in Poland have demonstrated a very worrying tendency for lipid disorders to occur in 70% and overweight and obesity in more than half of Poles (POLKARD, 2009). Yet, no such studies have been conducted in this field among the disabled, including people with vision impairment. Furthermore, few research centres have examined health status in this group. Previous publications have suggested higher morbidity rates for the blind and the visually impaired, including an increased incidence of cardiovascular diseases (Gapella-McDonnall, 2007; Jones, 2010). Bilyk et al. (2011) found that one of the factors inducing this problem is limited access to healthy food and an increased amount of ready-made highly-processed foods combined with a low level of physical activity. Regular involvement of blind and visually impaired people in sports is likely to reduce cardiovascular risk. Therefore, in the present study, we attempted to evaluate the lipid profile and nutritional status of male goalball players.

## Material and Methods

### Participants

Thirty two blind or visually impaired male goalball players aged 20 to 45 years participated in the study. The subjects were recruited from 5 teams in Poland. Inclusion criteria for participation in the study were as follows: severe or moderate disability caused by vision impairment (code H54 according to ICD-10) and involvement in athletic activity for at least 3 years. The subjects were not included in the study if they did not provide consent for participation. The examinations were carried out before a goalball tournament played in Poland championships in November 2014.

A direct observation methodology was employed with somatic variables and the lipid profile analysed. Among somatic variables, body height (BH), body mass (BM), weight circumference (WC as specified in WHO standards in 2008) and visceral fat rating (VFR) were measured. Fat rating was determined by means of a Tanita Viscan AB-140 visceral fat analyser. The BMI was calculated and compared with the standard levels adopted by WHO in 2007. The lipid profile was examined, including total cholesterol (TC), LDL cholesterol (LDL), HDL cholesterol (HDL) and triglycerides (TG), as specified in the standards of the American Heart Association (2010). The blood samples (5 ml) were taken from the subjects in the morning before the tournament, 12 hours after an overnight fast. Blood levels of the lipid profiles were measured in the analytical laboratory at the Academy of Physical Education in Katowice using diagnostic tests developed by Randox. The experimental procedures were approved by the Bioethics Committee of the Jerzy Kukuczka Academy of Physical Education in Katowice (resolution No. 9/2012).

### Statistical Analysis

Arithmetic means (*χ̄*), standard deviations (SD), minimum (min) and maximal (max) values, as well as 95% confidence intervals for somatic variables (BMI, WC, VFR) and components of lipid profiles (TC, HDL, LDL, TG) were calculated. The Kolmogorov–Smirnov test was used to verify the normality of distribution of the data. The relationships between somatic variables (BMI, WC, VFR) and lipid profile components (TC, HDL, LDL, TG) were examined using the Pearson’s correlation coefficient. For each parameter, the study participants were divided into two groups (“standard” and “above standard”). Differences in mean components of the lipid profile between the groups were determined using the Student’s t-test. The significance level was set at p<0.01, p<0.05.

## Results

Anthropometric and biochemical variables of the subjects are shown in Table 1.

**Figure 1:**
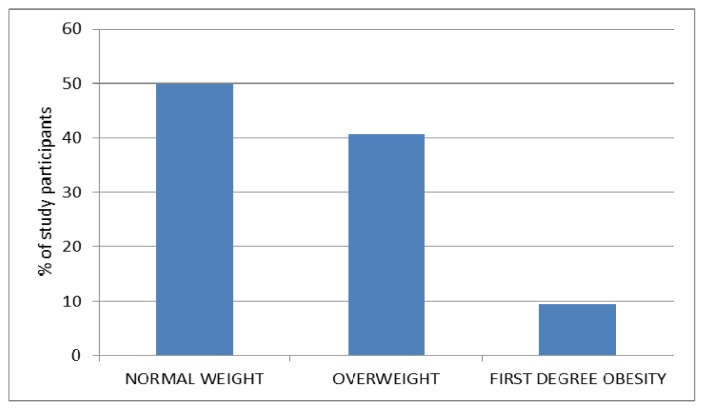
Percentage of study participants in individual BMI categories

Using the BMI criterion, overweight was found in 40.6% of athletes, with obesity being at the level of 9.3% (Figure 1).

Abdominal obesity expressed by waist circumference was observed in 43% of the study participants, whereas excess visceral fat was found in 28%. A high correlation was found between visceral fat and the BMI (r=0.7; p<0.001), as well as visceral fat and WC (r=0.8; p<0.001).

**Figure 2:**
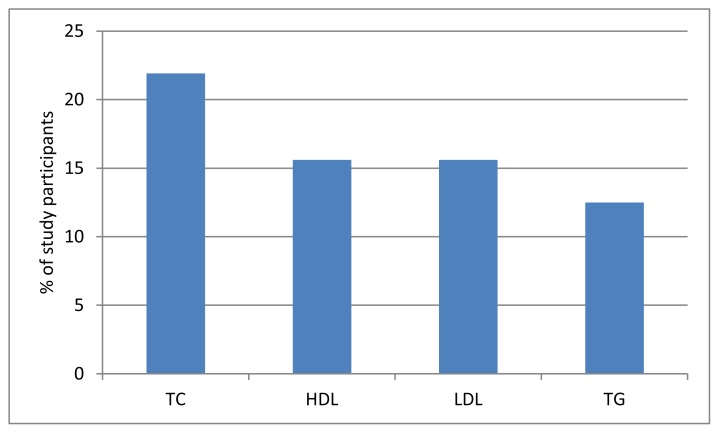
Prevalence of lipid disorders

Abnormal total cholesterol levels were recorded for LDL (22% of study participants), HDL (17%) and TG (13%) (Figure 2).

Lower levels of individual components of lipid profiles (and higher levels for HDL) were found in athletes with a normal BMI. Statistically significant differences were found for total cholesterol (p<0.001), LDL cholesterol (p<0.05) and triglycerides (p<0.05).

Similar relationships were observed for waist circumference, with lower contents of individual lipid profile components found in the participants with standard WC. Statistically significant differences were also shown for HDL cholesterol (p<0.05) and triglycerides (p<0.01).

Furthermore, we found low positive correlations between the BMI and triglyceride content (r=0.4, p<0.01) and moderate positive correlations between waist circumference and triglyceride levels (r=0.4, p<0.01). A statistically significant correlation was also observed between visceral fat and LDL cholesterol (r=0.4; p<0.05) as well as TG (r=0.5; p<0.001).

## Discussion

According to the World Health Organization, cardiovascular diseases are responsible for almost half of the deaths worldwide caused by noncommunicable diseases (Alwan, 2010). Despite the substantial progress in prevention of these diseases, the mortality rate in Poland continues to be very high (Szuba et al., 2011).

Epidemiological studies have shown that dyslipidemia, overweight and obesity represent independent cardiovascular risk factors. According to WHO (2000), obesity is currently the most frequent metabolic disease, reaching the prevalence of an epidemic and becoming of highest health concern in developed countries. Obesity is likely to cause cardiovascular diseases, including arterial hypertension, ischaemic heart disease, circulatory collapse or cerebrovascular accident (Zahorska-Markiewicz, 2009). The guidelines for prevention and treatment of dyslipidemia, overweight and obesity suggest that people should live less sedentary lifestyles and be more involved in moderate physical activity (Zahorska-Markiewicz, 2009; Kay, 2006; Lee, 2005).

Physical activity of blind and visually impaired people is usually at lower levels compared to the general population (Holbrook et al., 2009; Aslan et al., 2012; Willis et al., 2012), leading directly to overweight, obesity and lipid disorders (Holbrook et al., 2009; Steinman and Vasunilashorn, 2011). Regular involvement in sports is likely to reverse this negative tendency, which was partly demonstrated in our study. We found that 40.6% of goalball players were characterized by overweight and 9.3% by obesity. These indices were higher in a comprehensive study of Polish population, with the values of 48 and 32.8%, respectively (Jóźwiak, 2008). The percentage of men with normal weight was also higher (50 vs 18.8%). Higher indices of overweight and obese men were also found in surveys conducted by POLSCREEN (73%) and WOBASZ (67%) (Podolec and Kopeć, 2006; Broda et al., 2005). A statistically significant correlation was demonstrated between the BMI and triglycerides (r=0.38, p=0.03), which is in line with the findings of other authors (Jóźwiak, 2008).

Abdominal obesity was observed in 43% of the subjects. This number is higher compared to the male population in Poland since abdominal obesity was demonstrated in 40.2% of study participants. The increase in waist circumference was found to elevate triglyceride content (r=0.4; p<0.01). Waist circumference is correlated with visceral fat rating (Janssen et al., 2002) which is especially dangerous to human health. This fat represents an active secretory system that influences series of metabolic processes and several clinical surveys have shown its relationship with cardiovascular risk (Braszkiewicz, 2009; Carr, 2004; Skowrońska et al., 2005; Wajchenberg and Subcutanuous, 2000). Excess visceral fat was present in 28.1% of the subjects. Furthermore, a high positive correlation was found between visceral fat and (1) the BMI (r=0.7; p<0.000) as well as (2) waist circumference (r=0.8; p<0.01), which is consistent with the tendencies observed in the general population (Janssen, 2002). Body fat accumulation is attributable to synergistic effect of biological, behavioural, social and environmental factors, including chronic stress (Branca et al., 2007; Kyrou et al., 2006). Due to difficulties with everyday existence, blind and visually impaired people are often exposed to chronic stress, what may additionally contribute to accumulation of excess visceral fat, despite regular physical activity.

Cardiovascular risk is elevated for higher levels of cholesterol, its fractions and triglycerides. In our study, 21.9% of blind and visually impaired athletes were found to have elevated total cholesterol levels. This percentage was lower compared to the results obtained in surveys conducted for the general population in Poland by NATPOL 2011 (61%), POLSKREEN (65.1%) and WOBASZ (74%) (Zdrojewski et al., 2011; Podolec and Kopeć, 2006; Broda et al., 2005). Non-normative levels in the study participants were observed for LDL and HDL cholesterol (16.6%) as well as triglycerides (12.5%), with lower values than for the general population (55% for LDL, 21% for HDL, 23% for TG, respectively) (Broda, 2005). It can be expected that the most favourable lipid profile compared to the national-level survey was obtained for regular physical activity. Our findings are consistent with the results obtained in a study conducted within the NHANES program in 2003–2006, as physical activity was found to show a positive correlation with HDL cholesterol levels and a negative correlation with LDL and TG levels (Luke et al., 2001). A beneficial effect of physical activity on the lipid profile was also demonstrated in a study by Miller et al. (2011) and Komala et al. (2010).

The blind and visually impaired people often live sedentary lifestyles and eat unhealthy diets (Bilyk et al., 2007). Therefore, they are exposed to higher cardiovascular risk. A study by Jones et al. (2010) found 47.2% of blind and 43.1% of visually impaired subjects suffering from cardiovascular diseases, whereas this percentage in the group of healthy controls accounted for 28.6%. The percentage of overweight and obese men was also lower compared to the general population of men in Poland, with a more beneficial lipid profile. Regular physical activity of the study participants is likely to have a positive effect on their health. Low sample size and no reference to blind and visually impaired non-athletes suggest that more research is needed into the problems of this population, both athletes and those not involved in any sports.

## Figures and Tables

**Table 1:** Basic somatic and biochemical data of the study participants

Variable	Mean (*χ̄*)	Standard deviation (SD)	Minimum (min)	Maximum (max)	Confidence -95.00%	Confidence 95.00%
Age	29	9.03	20	45	25.74	32.26
BM [kg]	78.89	14.74	51.2	105.9	73.57	84.2
BMI [kg/m^2^]	25.32	4.16	18.1	38	23.82	26.82
WC [cm]	90.91	12.88	71	117	86.26	95.55
VFR	9.83	7.42	1	33.1	7.16	12.51
TC [mg/dl]	164.65	35.8	112.1	254.7	151.74	177.55
HDL [mg/dl]	51.57	15.54	26.01	100.9	45.96	57.17
LDL	91.48	28.27	52.59	149.25	81.28	101.67
TG [mg/dl]	108.02	51.86	46.35	264.91	89.32	126.72
